# Epileptogenic LGG surgery with seizure freedom purpose: Supratotal resection (ETT-SpTR) based on Electrocorticography and navigated transcranial magnetic stimulation

**DOI:** 10.1007/s00701-025-06636-y

**Published:** 2025-10-08

**Authors:** Francesca Battista, Giovanni Muscas, Andreea Cristina Aldea, Eleonora Visocchi, Alberto Parenti, Camilla Bonaudo, Maddalena Spalletti, Riccardo Carrai, Giulia Masi, Antonio Maiorelli, Andrea Amadori, Davide Gadda, Antonello Grippo, Alessandro Della Puppa

**Affiliations:** 1 Department of Neurosurgery, Department of Neuroscience, Psychology, Drug Area and Child Health (NEUROFARBA), University of Florence, Careggi University Hospital, Florence, Italy; 2 Neurophysiology Unit, Neuro-Muscular-Skeletal Department, Careggi Hospital, University of Florence, Florence, Italy; 3 Neuro-Anesthesiology and Intensive Care Unit, Careggi University Hospital, Florence, Italy; 4 Department of Neuro-Radiology, Careggi Hospital and University of Florence, Florence, Italy

**Keywords:** EPILEPSY, SURGERY, NTMS, LGG

## Abstract

**Background:**

Low-grade gliomas (LGG)—related seizures may persist after gross total resection (GTR). Supratotal resection (SpTR) seems to have better seizure outcomes, likely due to removing the epileptogenic peritumoral neocortex. However, its role in achieving postoperative seizure freedom remains poorly considered, likely because SpTR is achievable in only one out of three patients.

**Methods:**

We retrospectively analyzed a prospectively collected series of epileptogenic surgically resected LGGs. Intraoperative Electrocorticography (iECoG) guided the extension of GTR to areas with interictal activity and negative on navigated Transcranial Magnetic Stimulation (nTMS). Patients were divided into Group I (GTR) and Group II [iECoG nTMS Tailored – SpTR (ETT-SpTR)], and we compared the seizure outcomes at follow-up (minimum 12 months). We also compared the rate of postoperative neurological deficits.

**Results:**

Thirty patients were included. Group I (n = 15) showed only a 20% rate of seizure freedom (Engel IA), compared to 86.6% in Group II (n = 15, p = 0.0001). Neurological outcomes showed no differences between groups. Four patients (13.3%) with resection margins < 1 cm from nTMS-positive points developed transient deficits; no deficits were observed for distances > 1 cm.

**Conclusion:**

The ETT-SpTR is more frequently achievable than radiologically defined SpTR. In our experience, ETT-SpTR yields better seizure outcomes without compromising functional outcomes compared to GTR. In our cohort, iECoG is a reliable technique for identifying LGG-related epileptogenic foci, while nTMS is a trustworthy method for predicting postoperative deficits.

## Introduction

Epileptic seizures are a frequent symptom in patients with low-grade glioma (LGG) [35] and often persist even after gross total resection (GTR) [29]. The persistence of seizures significantly reduces patients'quality of life (QoL) due to the recurrence of critical events and their clinical and social consequences, as well as the cognitive effects of antiseizure medications (ASM). LGG patients are often young with long survival rates [15], and improving QoL should be an even more critical objective of surgical treatment for this patient group. Increasing evidence suggests that SupraTotal Resection (SpTR) offers a higher likelihood of achieving seizure freedom in LGG-related epilepsy [25, 37], likely because epileptic foci (EF) in the peritumoral neocortex are included in the resection. However, since LGGs are infiltrative tumors, SpTR is unachievable in 2 out of 3 cases due to the proximity of eloquent areas, with GTR often being the compromise to maintain the onco-functional balance.

In vitro, studies demonstrate that LGG-related EFs are located in the peritumoral neocortex micro-infiltrated by tumor cells, and epileptiform discharges originate from healthy neurons in these EFs as a result of changes in the extracellular microenvironment induced by tumor cells [30]. Consequently, GTR does not always include these areas in the resection, which explains the frequent persistence of seizures observed postoperatively [2, 29, 40, 47].

Our study aims to evaluate the efficacy and accuracy of Intraoperative Electrocorticography (iECoG) in identifying and localizing EFs and to assess whether including them in the surgical resection of the tumor improves the patients'epileptological outcomes. The second aim of our study is to evaluate how navigated transcranial magnetic stimulation (nTMS), validated through direct cortical stimulation (DCS), effectively predicts the functionality of the cortical area where the EFs are located to avoid postoperative neurological deficits. This approach enables a SpTR, which can be named SpTR tailored by iECoG and nTMS (ETT-SpTR).

## Material and methods

We retrospectively analyzed a series of prospectively enrolled patients with intracranial LGG and epileptic seizures as the onset symptom undergoing surgical resection at our center. We have considered epileptic seizures to be all those phenomena in which a clinical manifestation has been correlated with critical/intercritical electroencephalographic abnormalities.

### Preoperative MRI

All patients underwent magnetic resonance imaging (MRI) at our center using a 3 T Tesla MRI machine (Ingenia 3 T, Philips Medical Systems, Best, The Netherlands) with the standard oncological protocol [9]. Diffusion tensor imaging (DTI) sequences were carried out for white matter tract reconstruction.

### Preoperative navigated Transcranial Magnetic Stimulation (nTMS)

Preoperative functional cortical areas were defined using nTMS with a figure-of-eight coil (Galileo NetBrain Neuronavigator 9000, EB Neuro Corp., Florence, Italy). A volumetric T1-weighted MR image was used to build a patient's 3D brain model. We consider resting motor threshold (rMT) as the minimal TMS intensity to obtain a motor evoked potential (MEP) of 50 µV, registered at the right first dorsal interosseus (FDI) muscle, in about 50% of 5 to 10 consecutive trials [3, 39]. We adopted a monophasic pulse stimulation to investigate motor areas and a biphasic pulse stimulation with a fixed intensity above 110% of the rMT to test language and calculation function.

For mapping the motor cortex, we measured the amplitudes of the MEPs evoked in muscles contralateral to the stimulation.

The language task consisted of the D80-naming test, whereas the calculation task required simple single-step arithmetic operations. Before each repetitive TMS (rTMS) mapping, the patient performed the'baseline'task, i.e., completed without rTMS perturbation, to remove all incorrect answers from the stimulation sequence. Then, rTMS perturbation consisted of trains of 5 pulses at 5 Hz with an intensity of 110% of the FDI rMT, delivered simultaneously with the presentation of pictures, showing the D080 naming test for language or arithmetic operations for calculation [27]. For the language function, 90 trains were applied over the left perisylvian cortices, while the calculation function was mapped on 70 spots, distributed over parietal, temporal, frontal, and prefrontal cortices.

### Seizure preoperative management

We included patients with preoperative epileptic (interictal or ictal) activity at standard EEG. For scalp EEG recording, electrodes were positioned on the scalp according to the international 10–20 system [24] (Galileo NT line, EB Neuro Corp., Florence, Italy). A neurophysiology expert interpreted the EEG traces. Electrographic and electroclinical seizures were defined according to Hirsch's criteria [16]. Antiseizure therapy with Levetiracetam was initiated at a standard dosage of 500 mg twice daily [44]. After surgery, the Levetiracetam 1000 mg/day was continued for all the follow-up time.

### Intraoperative setting of ETT-SpTR

Preoperative MRI with DTI sequences and TMS data were loaded onto the Neuronavigation system (StealthStation 8, Medtronic, Dublin, Ireland), aiming to enable constant monitoring of the distance between functional structures and surgical resection margins during surgery.

Perioperative Levetiracetam bolus before surgical incision and after induction was not administered to observe intercritical activity on iECoG. After opening the dura, the anesthetic regimen involved Dexmedetomidine and Remifentanil, avoiding Propofol to prevent the suppression of the iECoG trace (burst suppression [5, 21, 22]).

**Fig. 1:**
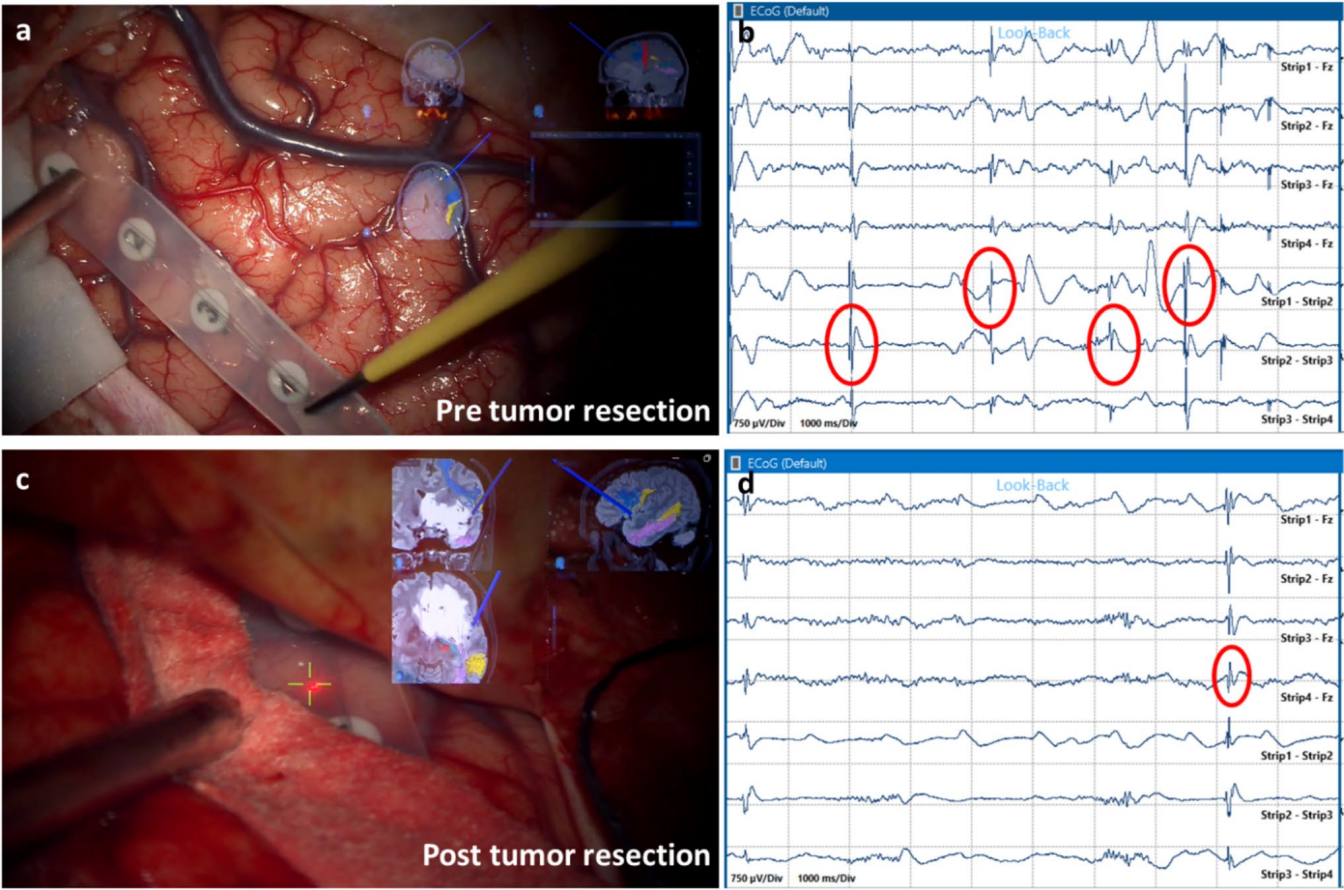
A case of fronto-basal LGG with a temporal extension. We reported the pre-resection iECoG registration: (a) the strip is positioned on the frontal lobe, in the superior margin of the tumor; (b) in frontal registration, we identified positive iECoG areas, with a high density of spikes (HREA) and we extended the resection to these frontal cortical areas; (c) after the tumor + HREA resection, we positioned the strip to the margin of the resection; (d) less interictal activity, almost disappeared, was detected during the postresection exploration, so we did not additionally extend the resection to these cortical areas. LGG: low-grade glioma; iECoG: intraoperative Electrocorticography; HREA: high-risk epileptogenic areas

We utilized Cz'-Fz, C3'-Fz, C4'-Fz, and C3'-C4'montages for intraoperative EEG recording. For iECoG, a six-contact subdural strip (Wyler electrodes, AD-Tech Medical Instrument Corporation, Oak Creek, WI, USA) was placed on the exposed cortical surface, and each contact was referenced to Fz. The iECoG strip was positioned in multiple exposed cortical areas along the planned GTR margins. iECoG recording was performed before, during, and repeated at the end of resection at the cortical point with a higher frequency of interictal activity. Figure 1 is an example of iECoG in pre- and post-ETT-SpTR.

The iECoG was assessed by an expert neurophysiologist throughout the recording to define interictal activity based on Hirsch's criteria [16]. The same neurophysiologist evaluated both the pre- and postoperative EEGs. He estimated the interictal activity detected in the iECoG by counting the interictal graph elements defined by Hirsch's criteria [16] for each recorded frame (as shown in Fig. 1, we have 10-s screens). We adopted this method to objectively define variations in iECoG frequency across different surgical phases. We extended the resection until the interictal activity decreased significantly, and after a few minutes, it almost disappeared. We considered a reduction to less than 50% of interictal graphoelements within a recording segment of at least 10 s as a significant reduction. The decision to include an iECoG area with epileptiform activity (high-risk epileptogenic area—HREA) in the resection margins was based on the risk of neurological deficits. Thus, the functionality of the cortical area was defined using nTMS, tractography, and DCS. We did not extend the resection to cortical areas positive to iECoG when these were functional or when iECoG did not show epileptiform abnormalities.

At the opening of the dura mater, we checked the localization of any nTMS positive point over the exposed cortical area with the Neuronavigation probe. We calculated the distance, classified as less than or greater than 1 cm, from any nTMS-positive points to the margins of exeresis. After this, we proceeded with DCS. The decision between awake and asleep surgery was based on the location of the LGG, tumor characteristics, and the patient's condition. We determined the language hemisphere dominance according to the nTMS [14]. When possible, we opt for awake surgery; in cases of relative contraindication to awake surgery, we perform asleep surgery [49].

In awake surgery, the Penfield technique employed low-frequency stimulation for motor, language, and calculation cortical mapping (0.5–3 mA) [34]. An expert Logopedic guided patients during stimulation.

In the case of asleep surgery, the DCS employed the Taniguchi paradigm, utilizing high-frequency stimulation to investigate only motor function and monitor with MEPs [41]. The language function was preserved using the positive and negative nTMS language points identified on the neuronavigator.

An expert neuroradiologist calculated the resection volume on early postoperative MRI to define the extent of resection (EOR) compared to the preoperative MRI based on the RANO criteria [46]. The EOR was calculated using a voxel-based system of the software 3D Slicer [23]. GTR was defined as the total removal of the abnormal hyperintense signal present in the FLAIR sequences on the preoperative MRI [37], and subtotal or partial resection was defined as the presence of a residual nodular FLAIR signal abnormality [28]. The ETT-SpTR was defined as the removal beyond the abnormal hyperintense signal present in the FLAIR sequences on the preoperative MRI, including HREA identified on iECoG and negative on nTMS. The definition of ETT-SpTR was based on postoperative MRI and the intraoperative findings. Cases of subtotal or partial resections were excluded from the study. Only cases that had undergone at least a GTR were included. Retrospectively and based on the EOR defined, we divided our study population into two groups: Group I, patients undergoing GTR of the glioma, the control group, and Group II, patients undergoing ETT-SpTR. After discharge, follow-up was conducted, including clinical and electroencephalographic monitoring, as well as radiological assessment. The Engel classification [10] was used to define epileptological outcomes at the 12-month clinical evaluation after surgery. The postoperative functional outcome was assessed by the percentage of neurologic deficits that worsened, improved, or remained unchanged postoperatively. We evaluated pre- and postoperative neurological status through the Aachener Aphasie Test (AAT) for language [18] and the Medical Research Council (MRC) scale for motor function [33]. We calculated the incidence of these deficits in both Group I and Group II. We assessed the statistically significant difference in percentages between the two groups using comparison tests and calculated the odds ratio (OR) and relative risk (RR).

Patients whose postoperative histological report did not confirm the suspicion of LGG, those without seizures confirmed by EEG, and those with partial surgical resection of LGG were excluded from the study, as were patients who died during follow-up.

### Statistical analysis

Quantitative measurement data were expressed as mean ± standard deviation (SD). We analyzed qualitative variables by summarizing them as frequencies and percentages, and relationships between variables were assessed using Fisher's exact test and the Chi-square test. Analysis of variance (ANOVA) or"N-1"Chi-squared test was used to assess statistical differences between the two groups or percentages. A p-value < 0.05 was considered statistically significant. ORs and RRs were calculated to assess the statistical significance of associations. ORs were used to measure the strength of association between categorical variables, while RRs were calculated to estimate the risk of an event in one Group compared to another. These methods were chosen to assess the significance of relationships between the analyzed variables accurately.

A binomial logistic regression analysis was performed, including relevant clinical covariates such as tumor location (temporal or extratemporal), histology (oligodendroglioma or astrocytoma), seizure semiology (focal or generalized), postresection iECoG (positive or negative), and surgical technique (ETT-SpTR vs GTR). We calculate the OR and the 95% confidence interval (CI) for each variable. We calculated the positive predictive value (PPV) and negative predictive value (NPV) of ETT-SpTR in relation to seizure outcome. These metrics were calculated to evaluate the clinical utility and diagnostic accuracy of the ETT-SpTR in predicting seizure outcomes. Additionally, we computed the receiver operating characteristic (ROC) curve, which is a graphical representation of the trade-off between sensitivity and specificity at various thresholds, to assess the diagnostic performance of the model. For the calculation of the ROC curve, we considered Engel IA as a good seizure outcome and Engel > IA as a negative outcome. The statistical analysis was conducted using IBM SPSS Software (IBM Corp., 2023. IBM SPSS Statistics for Windows, Version 29.0.2.0, Armonk, NY: IBM Corp).

## Results

We collected 35 patients between March and December 2023 diagnosed with LGG undergoing total resection, with seizures as the presenting symptom. We excluded 3 patients because of subtotal or partial resection, 1 patient because of a revision of the histopathological report with a new diagnosis of WHO grade III glioma, and 1 patient because he died during the follow-up. Therefore, we included 30 patients in our study (Table 1).

**Table 1:** Demographic and Clinical Characteristics of Patients with LGG undergoing GTR. LGG: low-grade glioma; GTR: gross total resection; SD: standard deviation; iECoG: intraoperative electrocorticography

Characteristic	Value
Total Patients	30
Gender	Male: 17 (56.6%) Female: 13 (43.46%)
Mean age (± SD)	46.4 years ± 17.02 (Range: 23 to 74 years)
Median Age	46 years
Mean follow-up (± SD)	16.43 months ± 3.09 (Range: 12 to 21 months)
Seizure Medication	Levetiracetam 500 mg twice daily
Lesion Location	Frontal: 10 (33.3%) Parietal: 5 (16.7%) Temporal: 15 (50%)
Lesion side	Left: 15 (50%) Right: 15 (50%)
Hemisphere dominance	Left 29 (96.6%) Right: 1 (3.4%)
Awake Surgery	6 (20%)
Asleep Resection	24 (80%)
Resection Side	Left: 15 (50%) Right: 15 (50%)
Positive iECoG	18 (60%)
Group I	15 (50%)
Group II	15 (50%)

**Fig. 2:**
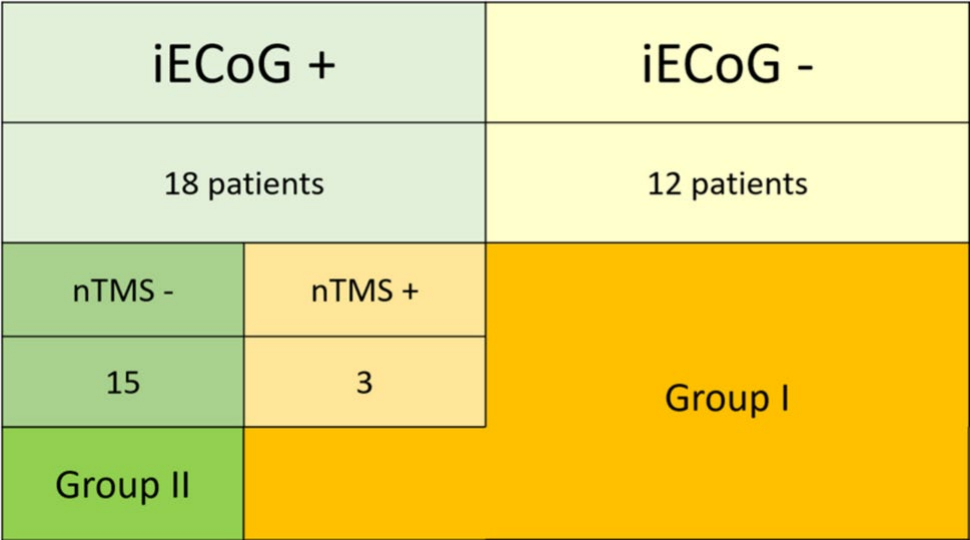
Schema of patient allocation Group, based on iECoG and nTMS data. iECoG: intraoperative Electrocorticography; TMS: navigated Transcranial Magnetic Stimulation

Seventeen were male, and thirteen were female (56.6% vs. 43.46%). The mean age was 46.4 years ± 17.02 (range 23 to 74 years) with a median of 46 years. The mean follow-up was 16.43 months ± 3.09 (range 12–21 months). All patients in the study initiated Levetiracetam 500 mg twice daily at the diagnosis of seizures and continued it throughout the entire follow-up period. Preoperative EEG traces showed interictal activity in all patients. In 10 patients, the lesion was located in the frontal region (33.3%); in 5, it was in the parietal region (16.7%); and in the remaining 15 cases (50%), it was in the temporal region. Resection occurred on the left side in 15 cases (50%) and on the right side in 15 cases (50%). We performed the awake surgery in 6 patients (20%) and asleep resection in the other 24 cases (80%). Thirteen oligodendrogliomas (1p-19q codeleted, WHO grade 2; 43.3%) and 17 astrocytomas (WHO grade 2; 56.7%) were diagnosed. The total number of patients with positive iECoG was 18 (60%): in 15 cases, we included the HREA in the resection, obtaining an ETT-SpTR, while in the remaining 3 cases, it was not possible to include the HREA in the resection because it was located in areas corresponding to functional cortical regions (nTMS positive). Therefore, these 3 patients were placed in Group I, i.e., those who achieved a GTR. The other 12 patients in Group I were cases where the iECoG was negative; thus, extending the resection to these undetected areas was impossible. Then, we included 15 patients in Group I (50%), 12 with negative iECoG and three with cortical point positive both to iECoG and nTMS, and 15 in Group II (50%) (Fig. 2). Table 2 reports demographic data, histopathology, and seizure data of the two Groups.

**Table 2:** Characteristics of the two study Groups

	**Group I**	**Group II**
Number of patients	15 (50%)	15 (50%)
Gender	7 Male; 8 Female	10 Male; 5 Female
Histopathology	- 7 Oligodendrogliomas (1p-19q codeleted, WHO grade 2) - 8 Astrocytomas (WHO grade 2)	- 6 Oligodendrogliomas (1p-19q codeleted, WHO grade 2) - 9 Astrocytomas (WHO grade 2)
Seizure type	All focal seizures	All focal seizures
Seizure semiology	- 10 Focal myoclonic seizures - 5 Dysautonomic seizures	- 9 Focal myoclonic seizures - 4 Sensory seizures - 1 Auditory hallucination
Seizure duration (Mean)	6.32 ± 0.4 months	5.45 ± 1.2 months

The multivariate logistic regression analysis assessed the independent association between clinical variables and seizure outcome. The model included tumor location, histology, seizure semiology, postresection ECoG usage, and surgical technique (ETT-SpTR vs. GTR). ETT-SpTR was identified as the only independent predictor of favorable seizure outcome (p = 0.009; Exp(B) = 0.000), suggesting a strong association with Engel IA. The remaining covariates did not reach statistical significance. Given the limited sample size and the observed extreme parameter estimates, the model should be interpreted cautiously (Table 3).

**Table 3:** Multivariate logistic regression analysis assessing predictors of favorable seizure outcome (Engel class IA). Among the included variables (tumor location, histology, seizure semiology, post-resection ECoG, and surgical technique), only ETT-SpTR was significantly associated with better seizure outcomes (p = 0.009). The model suggests a potential independent effect of ETT-SpTR; however, extreme confidence intervals reflect model instability due to sample size limitations

Variables	*p-*value	Odds ratio (OR)	95% Confidence Interval (CI)
Temporal vs extratemporal locations	0.527	0.414	[0.027–6.353]
Oligodendroglioma vs Astrocytoma	0.071	0.675	[0.038–12.090]
Focal vs generalized seizures	0.259	2.118	[0.118–38.001]
Positive vs negative post-resective ECoG	1.000	0.852	[~ 0—∞]
ETT-SpTR vs GTR	**0.009**	0.100	[~ 0—∞]

**Fig. 3:**
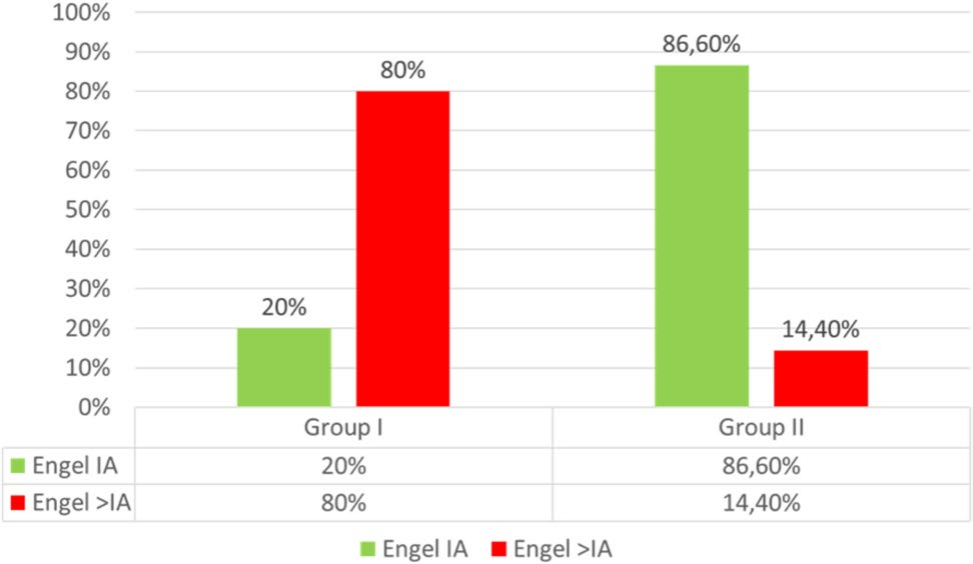
Graph showing the distribution of seizure outcomes in the two study groups: we achieved seizure freedom (Engel IA) in 86.6% of cases in Group II (ETT-SpTR) and 20% of cases in Group I (control), with a statistically significant difference (p = 0.0003). ETTSpTR: Electrocorticography and Navigated Transcranial Magnetic Stimulation Tailored SupraTotal Resection

In all Group II patients, interictal anomalies in the iECoG trace significantly decreased and almost disappeared after surgical resection. None of the Group I patients showed complete regression of interictal anomalies in postoperative scalp EEG. Thirteen out of 15 Group II patients (86.6%) remained seizure-free after surgery and during follow-up (Engel IA), while only three Group I patients (20% of Group I) had outcomes comparable to these (Engel IA); the remaining fourteen patients (46.7%) experienced epileptic seizures during follow-up (4 patients Engel IIA; 3 patients Engel IIIA; 2 patients Engel IIB; 4 patients Engel IC; 1 patient Engel IVB). Seizure control was achieved in 20% of Group I patients and 86,6% of Group II patients (p = 0.0003, OR = 0.0385 [95% CI: 0.01 to 0.27], RR = 0.16 [95% CI: 0.00 to 0.62]) (Fig. 3).

**Fig. 4:**
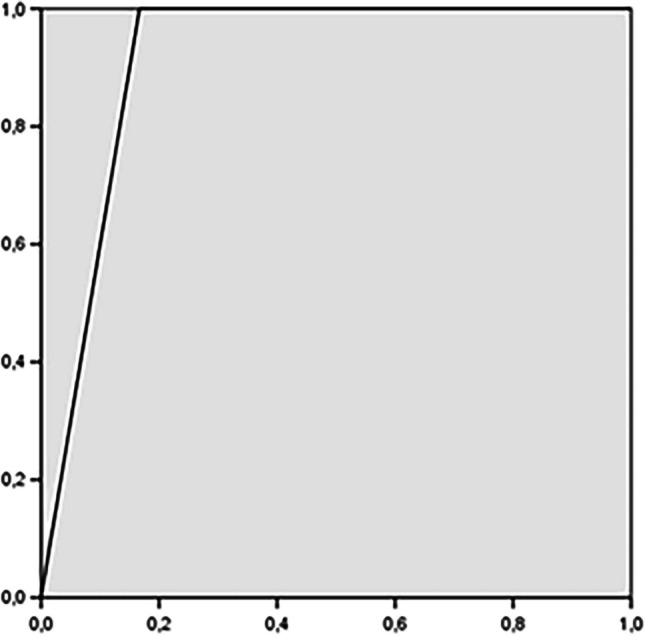
ROC curve: ETT-SpTR accuracy in predicting seizure outcome. The AUC is 0.917. ETT-SpTR: Electrocorticography and navigated Transcranial Magnetic Stimulation Tailored SupraTotal Resection; AUC: area under the curve

The PPV of ETT-SpTR on seizure outcome is 80%, and the NPV is 100%. The area under the curve (AUC) is 0.917 (Fig. 4).

In Group I, we observed the postoperative functional outcome: we noted 0 (0%) patients with improvement in neurological status, 13 (86.6%) patients with stability in neurological examination (no deficit before and after surgery), and two patients (13.3%) with worsening neurological deficit (1 mild aphasia 5/6 AAT, and 1 mild hemiparesis 4/5 MRC, both after surgery) In Group II, postoperatively, we observed one (6.6%) patient with neurological improvement of previously hemiparesis (before surgery MRC 4/5 and after surgery MRC 5/5), 12 patients (80%) with neurological stability (no deficit before and after surgery), and two patients (13.3%) with worsening neurological status (both with mild aphasia 5/6 AAT after surgery). The difference between the two groups did not show statistical significance (p = 1, OR = 1 [95% CI: 0.12–8.21], RR = 1 [95% CI: 0.16–6.20]) (Table 3) (Table 4).

**Table 4:** Postoperative Outcomes in Group I and Group II Patients

Outcome	Group I	Group II
Neurological Improvement	0 (0%)	1 (6.6%)
Neurological Stability	13 (86.6%)	12 (80%)
Neurological Worsening	2 (13.3%)	2 (13.3%)

We performed nTMS and verified the results with DCS in all patients. After opening the dura mater, we exposed 48 positive nTMS points in all 30 patients. Twenty of these 48 (40%) were positive motor points, 16 were positive language points (32%), 10 were positive calculation points (20%), and two were positive somatosensory points (4%). We found a distance of less than 1 cm in only four patients (8.3%), the same individuals in whom we observed a new postoperative neurological deficit. In all of these cases, the neurological deficit was transient, with complete recovery of functions during the follow-up. No postoperative deficit was recorded in patients where the distance of positive nTMS points was greater than 1 cm from the surgical resection margins.

## Discussion

Our study demonstrates the effectiveness and safety of the ETT-SpTR in improving seizure outcomes in our surgical series. We showed the effectiveness of iECoG in identifying EFs associated with LGGs intraoperatively and how their inclusion in surgical tumor resection improved the epileptological outcome in our series. nTMS has enabled the prediction of postoperative functional deficits with reasonable accuracy.

In recent decades, advancements in neuro-oncological treatments have increased the OS of patients with LGG to 15–17 years [4, 13]. In light of this, the neurosurgeon must aim for surgical resection goals that maximize radicality and achieve the best possible QoL for these patients. This need has been addressed by avoiding functional neurological deficits through surgical resection for years, thereby preserving the connectome [7]. However, in addition to functional outcomes, consideration must also be given to the control of epileptic seizures.

One of the most well-known definitions of SpTR was provided by Duffau and his group in 2016, describing SpTR as"the resection of the entire FLAIR-hyperintense lesion along with surrounding non-eloquent tissue, aiming to improve not only seizure but also oncological outcomes."[8] As this widely accepted definition goes beyond the traditional purely radiological description of SpTR for LGGs [37], it does not explicitly address how to identify the additional cortical areas to be included in the resection beyond those FLAIR-positive, despite emphasizing the need for these areas to improve oncological and seizure outcomes. In light of this gap, we conducted a study to investigate the current state of the art in molecular characterization of the peritumoral cortex. Several preclinical studies investigated the link between epileptogenesis and oncogenesis of LGGs [11, 43]. In vitro and pathological studies demonstrate that LGG-related epileptogenesis (EFs) is localized in the peritumoral neocortex and shares common molecular mechanisms with tumoral progression [30]. In particular, epileptogenesis is born from the interaction between glioma cells and healthy neurons of the peritumoral neocortex [11]. Glutamatergic excitatory mechanisms and GABAergic activity triggered by tumor cells generate epileptic activity in the neurons of the neocortex surrounding the gliomas [6, 32]. The alteration of glutamate transporter expression in tumor cells leads to an accumulation of extracellular glutamate, promoting epileptogenic discharges and tumor proliferation. This mechanism is exacerbated in cases where a mutation in the isocitrate dehydrogenase 1 (IDH1) gene occurs in LGGs, leading to the accumulation of D-2-hydroxyglutarate, a stereoisomer of glutamate [19].

Additionally, intracellular chloride dysregulation in glioma cells induces mitosis and proliferation while causing aberrant depolarization of GABA receptors in adjacent healthy neurons, thereby triggering epileptogenic discharges [32]. At the same time, this glutamatergic activity is believed to underlie the mechanism of tumor proliferation and infiltration into healthy parenchyma. Similarly, the mTOR pathway promotes both epileptogenesis and tumor proliferation and progression [19]. All these mechanisms are localized in the peritumoral cortex [17, 43, 45]. Therefore, with minimal microscopic tumor infiltration, tumor cells can induce epileptiform discharges in the healthy neurons of the peritumoral neocortex [26, 31, 36, 42]. These experimental findings are confirmed by intraoperative electrophysiological investigations using iECoG and ex vivo electrophysiological explorations of epileptiform electrical activity in the peritumoral neocortex [12].

Currently, there are few studies evaluating the clinical benefits in terms of seizure outcomes following surgical removal of the peritumoral neocortex with interictal activity in LGGs [1, 48]. Yao et al. show that using iECoG can identify the temporal pole involvement in the EF of temporal lobe LGG [48]. The authors studied only the temporal pole in selected cases of LGG using the iECoG, which included the temporal lobes. They focused their attention on surgical strategies, avoiding explanation of the benefit gained from the inclusion of non-tumoral cortex in LGG resection. However, they obtained similar results to ours, but their study was limited to temporal LGG. The demonstration of the effectiveness of the ETT-SpTR strategy for both temporal and extratemporal LGG confirms the prior hypothesized localization of EF in the peritumoral neocortex [29], independent of the localization of the tumor.

The EFs are thus located in the peritumoral cortex, in the outermost regions infiltrated by the tumor [43]. Tumor infiltration is typically visible on advanced MRI sequences, such as FLAIR and T2-weighted sequences, as areas of signal hyperdensity [37], and the actual definition of SpTR is based on these MRI's abnormality. It has been demonstrated that MRI-tailored SpTR correlates with better control of postoperative seizures, probably due to the inclusion in the resection of the EFs [37]. Nevertheless, such MRI-tailored SpTR is often not achieved in LGG patients. The retrospective surgical series published by Rossi et al. demonstrated that SpTR was pursued in 32.3% of cases among 449 patients [37]. A systematic review by Kreatsoulas et al. found that among 1301 patients with LGG, 29.0% underwent SpTR [25]. These studies indicate that, although SpTR is superior regarding OS [38], it remains feasible in a smaller percentage of cases.

The reason for this limitation in applicability likely lies in the MRI-tailored SpTR definition. SpTR is defined based on the radiological images of LGG and refers to removing all brain parenchyma corresponding to the signal abnormality observed in T2-weighted and FLAIR sequences of MRI [37]. Such SpTR is not always feasible, especially for LGGs, which infiltrate adjacent healthy and functional brain tissue. The hyperintense signal on FLAIR sequences represents both the parenchyma infiltrated by tumor cells, which should be included in the SpTR, and the peritumoral vasogenic edema, which is not infiltrated and should not be included in the SpTR. Consequently, FLAIR does not differentiate between these two areas, exposing the patient to the risk of removing functional regions not infiltrated by the tumor. This definition is imaging-based and lacks intraoperative functional data. For this reason, the neurosurgeon prefers not to remove the functional areas even if they may be included in the FLAIR abnormal signal, resulting in not achieving SpTR. Therefore, a new definition of SpTR is urgently needed to differentiate more effectively between areas that are truly micro-infiltrated by the tumor and those with only vasogenic edema, enabling SpTR to be achieved in a higher percentage of cases while limiting the risk of functional deficits.

In our surgical series, thanks to ETT-SpTR, we achieved SpTR in 50% of patients, or one in two. Therefore, the ETT-SpTR technique we proposed appears to allow for extending resection to SpTR in a higher percentage of patients than reported in the literature for radiologically defined SpTR, namely 29%5, despite the limited number of patients in our surgical series, which does not allow for a clear statistical comparison. ETT-SpTR achieved an epileptological outcome comparable to that of radiologically defined SpTR4 (86.6% vs. 91.7%), but was achieved in a greater number of patients. Our work is preliminary, but it demonstrates how adopting a new definition of SpTR (ETT-SpTR) may allow for greater radicality in more patients compared to the radiological definition of SpTR, and we attribute this improvement to more reliability of the iECoG than the abnormal FLAIR signal in the decision process of which brain parenchymal area are infiltrated by the tumor.

nTMS played a crucial role in the decision-making process. The ability to preoperatively assess the relationship between the tumor and functional areas allowed for a change in the surgical approach and the extent of the craniotomy, thus providing greater safety. Additionally, it made it possible to adequately and reliably inform the patient before surgery about the risk of not achieving a SpTR and the potential for persistent epileptic seizures in the postoperative period. Furthermore, the decision to include iECoG-positive areas in the resection was largely sustained using the nTMS data. In the presence of nTMS-negative points, the neurosurgeon proceeded with greater confidence and more frequently included these cortical areas in the resection, which might help achieve a higher percentage of patients undergoing SpTR.

The rate of postoperative deficits in our surgical series was comparable between the two Groups, suggesting the safety of ETT-SpTR compared to GTR. Among all the patients in the study, a 13.3% rate of transient postoperative deficits and 0% of permanent postoperative deficits were observed. Recent literature shows that in SpTR for intracranial LGG, the risk of transient postoperative deficits is 31.08%, and the risk of permanent deficits is 2.7%^20^. When comparing our results to the literature, it appears that the rate of permanent deficits is in line with the literature findings (p = 0.36), while the rate of transient deficits is statistically significantly lower than that reported in the literature (p = 0.04). This analysis suggests that our results are reliable and that the ETT-SpTR technique was safe in our experience.

## Conclusion

ETT-SpTR provided a better epileptological outcome than GTR in our surgical series, and it appears to be a safe technique that allows for a higher rate of Engel IA, based on our experience. iECoG with cortical strips is a good tool for identifying LGG-related epileptogenic foci in our series. nTMS predicted postoperative functional outcomes with good reliability and allowed for a more extended resection in our series, including EFs, even if they are close to functional cortical areas. ETT-SpTR was more frequently achievable in LGG patients than radiologically defined SpTR in our series, likely due to the combination of iECoG and nTMS, which provides reliability and safety to the surgical strategy. Further studies are needed to confirm these findings.

## Data Availability

No datasets were generated or analysed during the current study.
